# Towards the implementation of law n. 219/2017 on informed consent and advance directives for patients with psychiatric disorders and dementia. Physicians’ knowledge, attitudes and practices in four northern Italian health care facilities

**DOI:** 10.1186/s12910-023-00997-8

**Published:** 2024-01-06

**Authors:** Corinna Porteri, Giulia Ienco, Mariassunta Piccinni, Patrizio Pasqualetti

**Affiliations:** 1 Bioethics Unit, IRCCS Istituto Centro San Giovanni di Dio Fatebenefratelli, Via Pilastroni, 4, Brescia, 25125 Italy; 2 Department of Political Science, Law and International Studies – SPGI, Università di Padova, Padua, Italy; 3 Department of Public Health and Infectious Diseases, Section of Medical Statistics, Sapienza Università di Roma, Rome, Italy

**Keywords:** Law n. 219/2017, Informed consent, Shared care planning, Advanced care planning, Advance directives, Psychiatry, Dementia

## Abstract

**Background:**

On December 2017 the Italian Parliament approved law n. 219/2017 “Provisions for informed consent and advance directives” regarding challenging legal and bioethical issues related to healthcare decisions and end-of life choices. The law promotes the person’s autonomy as a right and provides for the centrality of the individual in every scenario of health care by mean of three tools: informed consent, shared care planning and advance directives. Few years after the approval of the law, we conducted a survey among physicians working in four health care facilities specific for the care of people suffering from psychiatric disorders, cognitive disorders and dementia located in the North of Italy aiming to investigate their perceived knowledge and training need, attitudes regarding law n. 219/2017 provisions, and practices of implementation of the law.

**Methods:**

A semi-structured questionnaire was developed on an online platform. The invitation to participate in the survey was sent by email to the potential participants. Information was collected by means of the online platform (Google Forms) which allows to export data in a spreadsheet (Windows Excel) to perform basic statistical analysis (frequency distributions, bar chart representation).

**Results:**

Twenty-five out of sixty physicians participated in the survey. None of the respondents value their knowledge of the law as very good, 10 good, 13 neither poor nor good, 1 poor and 1 very poor. All the respondents want to learn more about the law (21 yes and 4 absolutely yes). The majority of respondents agrees with the content of the law as a whole (3 absolutely agree, 13 agree), and on each provision. The question on the clarity of the concept of capacity in the law received mixed answers and this impacted on the physicians’ opinion regarding the legitimacy in principle for our groups of patients to realize shared care planning and write advance directives. Thirteen physicians neither introduced the theme of shared care planning nor arranged for shared care planning and the main reason for this was that no patient was in a clinical situation to require it. When shared care planning is realized, a variability in terms of type and number of meetings, mode of tracking and communication is registered.

**Conclusions:**

Our survey results indicate a need for more clarity regarding the interpretation and implementation of the law in the patient groups under study. There are in particular two related areas that deserve further discussion: (1) the question of whether these patient groups are in principle legitimized by the law to realize shared care planning or write advance directives; (2) the notion of capacity required by the law and how this notion can be declined in real-life situations.

**Supplementary Information:**

The online version contains supplementary material available at 10.1186/s12910-023-00997-8.

## Introduction

On 22 December 2017 the Italian Parliament passed law n. 219/2017 entitled “Provisions on informed consent and advance directives” [1], concerning pivotal and contentious healthcare issues such as consent and refusal of treatment, treatment withdrawal, advanced care planning, end-of-life decisions. The enactment of the law arrives after almost 30 years of a heated social and political debate and several failed legislative proposals [2, 3] and represents the final attempt to conciliate different and even conflicting moral beliefs and cultural positions about self-determination in healthcare and human life availability [4]. The need for a national regulatory provision was also made clear by some relevant judicial cases, such as those of Piergiorgio Welby, Eluana Englaro and Fabiano Antoniani. Moreover, law n. 219/2017 gives effect to international and supranational legislation, in particular the European Convention on Human Rights, the Oviedo Convention on Human Rights and Biomedicine and the Charter of Fundamental Rights of the European Union (whose articles 1-2-3 are directly recalled in the law text) [5].

The law provisions are grounded in the principles established by the Italian Constitution in articles 2, 13 and 32 - which affirm and protect inviolable human rights, personal freedom and the right to health: they aim to safeguard the person’s right to life, health, dignity and self-determination at all times of life, even in situations where the individual is temporarily or no longer able to express preferences and choices about medical treatments [6].

Law n. 219/2017 promotes the person’s autonomy as a right and provides for the centrality of the individual in every scenario of health care [7]. This is reflected in the way patient-physician interaction is understood and implemented [8]. According to the law provisions, the care relationship has to be characterised by continuous, bi-directional communication between the assisted person and the health-care providers, based on information sharing and valued by the appropriateness of the time spent with the patient: indeed, the doctor/patient communication is expressly understood as a critical component of the time of care [9]. Trust is also regarded as a fundamental component of the relationship, providing for the respect of both the patient’s autonomy and self-determination, and the doctor’s professionalism and expertise [10]. The law provisions value the concepts of appropriateness and proportionality of care, in accordance with the principle that the purpose of the therapeutic relationship is to realize a beneficial care, i.e. a care tailored to the individual person and achieved through the cooperation of the physician’s professional expertise and the patient’s interest in her/his care [11]. To improve this outcome, law n. 219/2017 also values the involvement of the patient’s family members and social relations [12].

Law n. 219/2017 finally gave a legal shape to the principles it states, especially by mean of three tools: informed consent (IC), shared care planning (SCP) and advance directives (AD), in the Italian law text respectively “consenso informato”, “pianificazione condivisa delle cure” and “disposizioni anticipate di trattamento”.

As a concise overview of the law provisions: article 1 (Informed consent) establishes the right of the patient to receive complete information regarding his/her health conditions -or, conversely, not to be informed and to delegate health-care decisions-, to give or refuse consent to medical treatments, to withhold consent to unwanted therapies, including life-sustaining ones. Article 2 (Pain therapy, prohibition of unreasonable obstinacy in treatment and dignity in the end of life) guarantees the patient’s right to appropriate pain therapy and provides for the medical doctor’s duty to avoid unreasonable obstinacy and unnecessary or disproportionate treatment in end-of-life care. Article 3 (Minors and incapacitated adults) includes specific provisions for minors and incapacitated adults and states their right to the valorisation of their capacity to understand and make choices. Specifically, the article regulates the expression of consent by persons who have not yet reached legal capacity because of their minor age and by those who are subject to a measure restricting their legal capacity according to the rules of the Italian Civil Code and specifies powers and duties of the legal representatives for these subjects. It is noteworthy that the Italian Civil Code (art. 404 ss.) provides for a case-by-case appointment by the court of a legal supporter for adult person who lack ability to provide to their personal interests, also taking into account their de facto capacity and clinical expert opinion about their ability to cope in daily life. The judgement of capacity and the configuration of the measure of legal support depend on the judge’s evaluation. Article 4 (Advance directives) states the citizen’s right to express wills and provide instructions on medical treatments in anticipation of a possible future incapacity for self-determination. Article 5 (Shared care planning) allows patients suffering from a chronic and disabling disease or disease characterized by an inevitable progression with unfavourable prognosis to collaboratively define a care plan with their physicians. The shared plan can be updated according to the evolution of the patient’s needs and health providers are obliged to comply with it if the patient becomes unable to give consent or enters a state of incapacity. The expression “advance care planning” (ACP) is commonly used in international language to refer to this process. The Italian wording emphasizes the collaborative nature of the process. Regulations on AD and SCP also include the individual’s right to nominate a trusted person (in the Italian law “persona di fiducia”/ “fiduciario”) with power of representation with healthcare professionals and organizations.

According to article 1 health facilities also maintain the obligation to ensure the full and correct implementation of the law principles, providing information for patients and training for the staff [13].

Few years after the entry into force of law n. 219/2017, on January 31st, 2018, the present survey aims to investigate perceived knowledge, attitudes and practices over the law provisions from the perspective of clinicians, with particular reference to psychiatrists, geriatricians, and neurologists working with patients suffering from psychiatric disorders, cognitive disorders and dementia. The importance, including social importance, of collecting data on how the law is implemented in different contexts is also referred to in art. 8 (Report to the Chambers).

Due to the specificity of our target patients’ disorders, the challenges of the care relationship - covered by the three elements of IC, SCP and AD - can be even more complex. The possibility of making health related choices is in fact dependent on the subjects’ competence, i.e. the ability to understand, appreciate, reason and make choices [14]. These abilities may be variously impaired in persons with psychiatric and cognitive disorders, although the diagnosis itself cannot predict competence and patients may have the ability to make medical decisions in the context of their illness, which makes the evaluation of competence a crucial and difficult task for physicians [15, 16]. Moreover, barriers to the implementation of ACP and AD particularly in these patient groups, including the difficulty of health-professionals to talk about the issue, have been recognized [17–20]. Across law n. 219/2017 the term ‘capacity’ is used non-homogeneously, sometimes to mean legal capacity other times to mean natural capacity (i.e. the de facto capacity to make decisions). This can make the reading of the law quite challenging. In addition, the law subordinates the expression of the right to self-determination to traditional capacity requirements (legal capacity and natural capacity) which are, however, insufficient to describe the complexity of the clinical conditions in which people may find themselves, especially if affected by psychiatric or cognitive disorders [21].

## Methods

### Survey

A questionnaire-based survey was conducted among physicians engaged in the care of people suffering from psychiatric disorders, cognitive disorders and dementia, aiming to investigate their perceived knowledge and training need, attitudes regarding law n. 219/2017 provisions, and practices of implementation of the law. An invitation letter providing a brief illustration of the study design and scope with the request to participate in the research by filling in an online questionnaire was sent by email to the potential participants by the principal investigator, who is the person responsible for the Bioethics Unit at IRCCS Fatebenefratelli (CP). The medical directors of the facilities were informed of the study, and they supported the initiative. For convenience, a copy of the law text was also attached to the email. Participation in the survey was voluntary and anonymous. The survey was conducted between October and November 2021.

The IRCCS (Italian Institute for Research and Care) Fatebenefratelli Ethics committee gave a favourable opinion on the study (opinion n. 76/2021).

### Questionnaire

A semi-structured questionnaire consisting of a total of 116 questions − 76 closed and 40 open-ended and optional aimed at deepening the closed answers - was developed for the present study by the authors (CP wrote the first draft; PP and MP gave essential contributions). Depending on the answer, a number of questions allowed some of the subsequent questions to be skipped. The questionnaire was organised into the following sections: (i) perceived knowledge of the law and need for further information and training; (ii) degree of agreement with the law as a whole and with each provision; (iii) theme of the subject’s capacity/incapacity; (iv) opinion on the legal legitimacy of SCP and AD for patients with psychiatric disorders, cognitive disorders, mild-moderate dementia; (v) opinion on the utility of SCP and AD for patients with psychiatric disorders, cognitive disorders, mild-moderate dementia; (vi) opinion on the importance of the trusted person; (vii) personal experience as clinician over SCP; and (viii) personal experience as clinician over AD.

Essential information (all optional) regarding doctors taking part in the survey, including their religious belief, was also collected. Information was not such as to make the subjects identifiable.

The first version of the questionnaire was submitted to three clinicians - a neurologist, a psychiatrist and a medicolegal doctor - to collect their comments on the content and formulation of the questions. We received few suggestions on the language of the questions to improve clarity (e.g. to include the clause “in principle” in the question on the legal legitimacy for our patients to realize SCP or write AD). Suggestions from the Ethics committee that approved the research protocol were also taken into consideration. The revised questionnaire was transferred to an online platform and, after the test of the platform functionality, used in the survey. [The questionnaire is available as Additional file]

### Participants

The questionnaire was sent to the medical doctors working in the four catholic health facilities specific for the care of people with psychiatric and cognitive disorders located in the North of Italy belonging to the same religious province of the survey promoter, the St. John of God Fatebenefratelli Order, to collect data as a first step of an action aimed at promoting education and better clinical practices if needed.

All the psychiatrists/physicians specialized in clinical psychology, geriatricians, and neurologists were invited to participate: they were 60 in total, of whom 34 were psychiatrists, 3 clinical psychologists, 15 geriatricians, 8 neurologists. The invited clinicians were not randomly selected and thus cannot be considered a representative random sample of Italian physicians engaged in the care of people suffering from psychiatric disorders, cognitive disorders and dementia.

### Data analysis

Information was collected by means of an online platform (Google Forms) which allows to export data in a spreadsheet (Windows Excel) to perform basic statistical analysis (frequency distributions, bar chart representation). Given the very few answers to the open-ended questions, no specific analysis was used. Two of the Authors with expertise in bioethics (CP) and methodology and statistics (PP) executed data analysis. [The dataset with responses to the questionnaire is available as Additional file]

Since the number of doctors was small, we avoided the use of percentages and reported absolute frequencies. In fact, the use of percentages, even for descriptive purposes, is not recommended for small sample sizes (“never compute a percentage unless the number of cases on which the percentage is based is in the neighbourhood of 50 or more” [22]).

## Results

In total, 25 clinicians participated in the survey (42% of the sample): 17 (out of 37) psychiatrists/clinical psychologists, 6 (out of 15) geriatricians and 2 (out of 8) neurologists. Descriptively the participation of psychiatrists/clinical psychologists and geriatricians was higher than the other group. Participants reported a time for completing the questionnaire between 10 and 40 min.

Fifteen of the respondents had more than 20 years of experience, 6 from 11 to 20 years, 1 from 6 to 10, and 3 from 0 to 5. Twenty declared themselves catholic christians, 1 non-catholic christian and 4 agnostics/atheists.

Below we present the study results (frequency distributions) following the survey sections and then we report few suggestions coming from the open-ended questions.

**Fig. 1:**
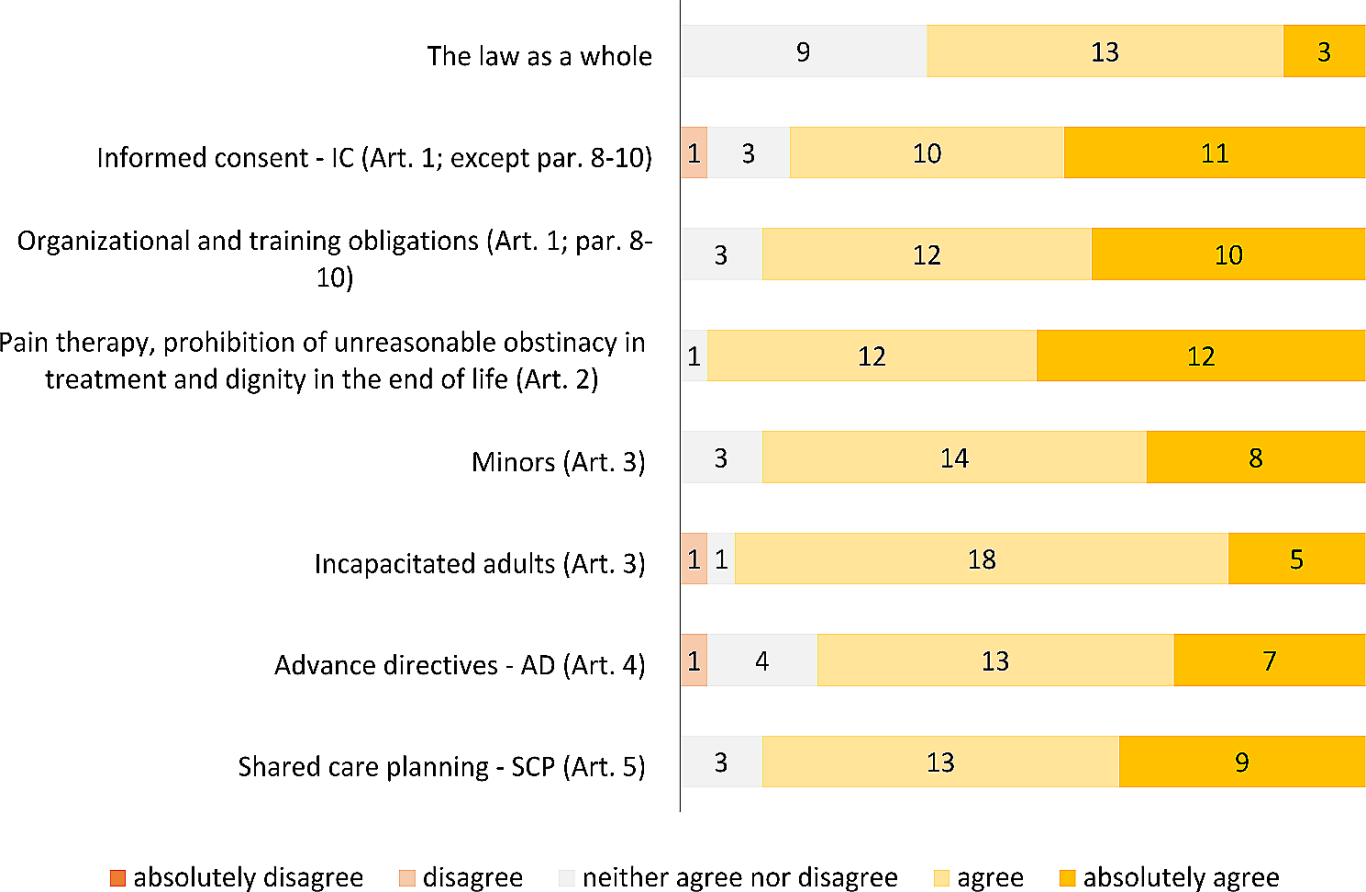
Physicians’ agreement on the law provisions

**Fig. 2:**
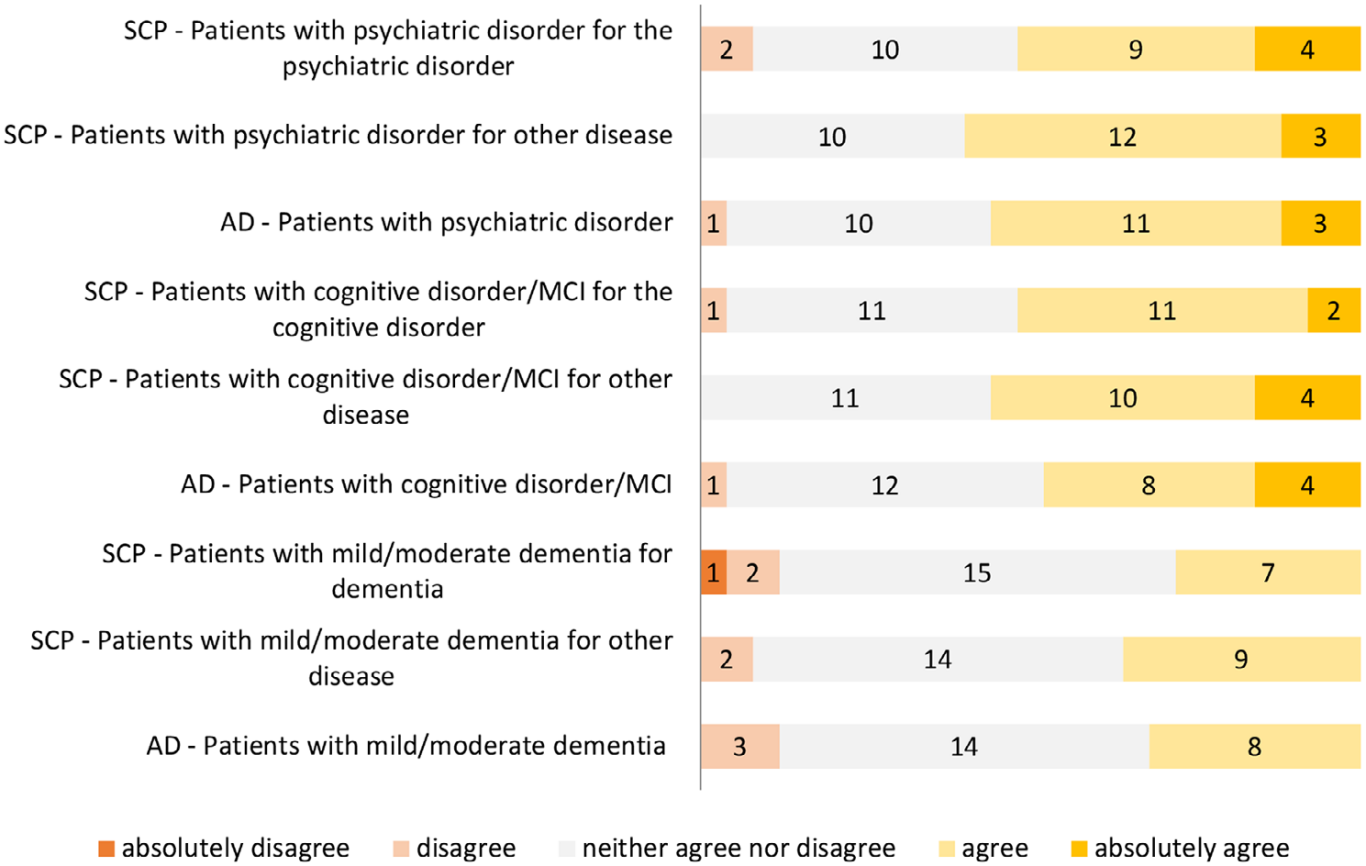
Physicians’ agreement on the legitimacy in principle of realizing SCP and writing AD in the three patient groups

i) Knowledge of the law and need for further information and training. None of the respondents value their knowledge of the law as very good, 10 good, 13 neither poor nor good, 1 poor and 1 very poor. The first source of information and training on the law was internet (13), followed by training organized by healthcare facilities (11, seven of which organized by their reference facility), scientific journals (5), mass media (4), and training promoted by non-health organizations (3). All the respondents want to learn more about the law (21 yes and 4 absolutely yes). The aspect of the law that all want to deepen (8 absolutely agree and 17 agree) is the role of the trusted person and family members. However, the participants would also like to deepen all the other aspects of the law, especially AD (13 absolutely agree, 10 agree), incapacitated patients and palliative care (12 absolutely agree, 11 agree), SCP (10 absolutely agree and 13 agree), communication with the patient and IC (8 absolutely agree, 15 agree). Among doctors who do not express agreement in exploring the issues above, no one explicitly disagrees (the neutral response was always chosen). Respondents are quite less interested in investigating the topic of minors, with 8 strongly agree, 6 agree, 9 neutral, and 2 disagreeing. This is likely due to the type of the target facilities that work with adults.

ii) Degree of agreement with the law as a whole and with each provision. The majority of respondents agrees with the content of the law as a whole (3 absolutely agree, 13 agree), 9 neither agree nor disagree and no one disagree. The level of agreement/disagreement on each article of the law is reported in Fig. 1.

iii) Theme of the subject’s capacity/incapacity. The question on the clarity of the concept of capacity in the law received mixed answers: we asked the participants if, as physicians specialized in psychiatric/cognitive disorder/dementia, they felt that the law adequately addresses the issue of the subject’s capacity/incapacity in view of a clear application in the clinical practice. Twelve respondents agree on the clarity of the law (no one absolutely agree); 9 neither agree nor disagree; 3 disagree and 1 absolutely disagree.

iv) Opinion on the legal legitimacy of SCP and AD for patients with psychiatric disorders, cognitive disorders, mild-moderate dementia. The respondents’ opinions on the legal legitimacy for patients with psychiatric disorders, cognitive disorders and dementia of realizing SCP for their psychiatric/cognitive disorder/dementia or eventually for other disease that is chronic and disabling or characterized by an irreversible progression with an unfavourable prognosis, and on the legal legitimacy for these patients of writing AD are reported in Fig. 2.

**Fig. 3:**
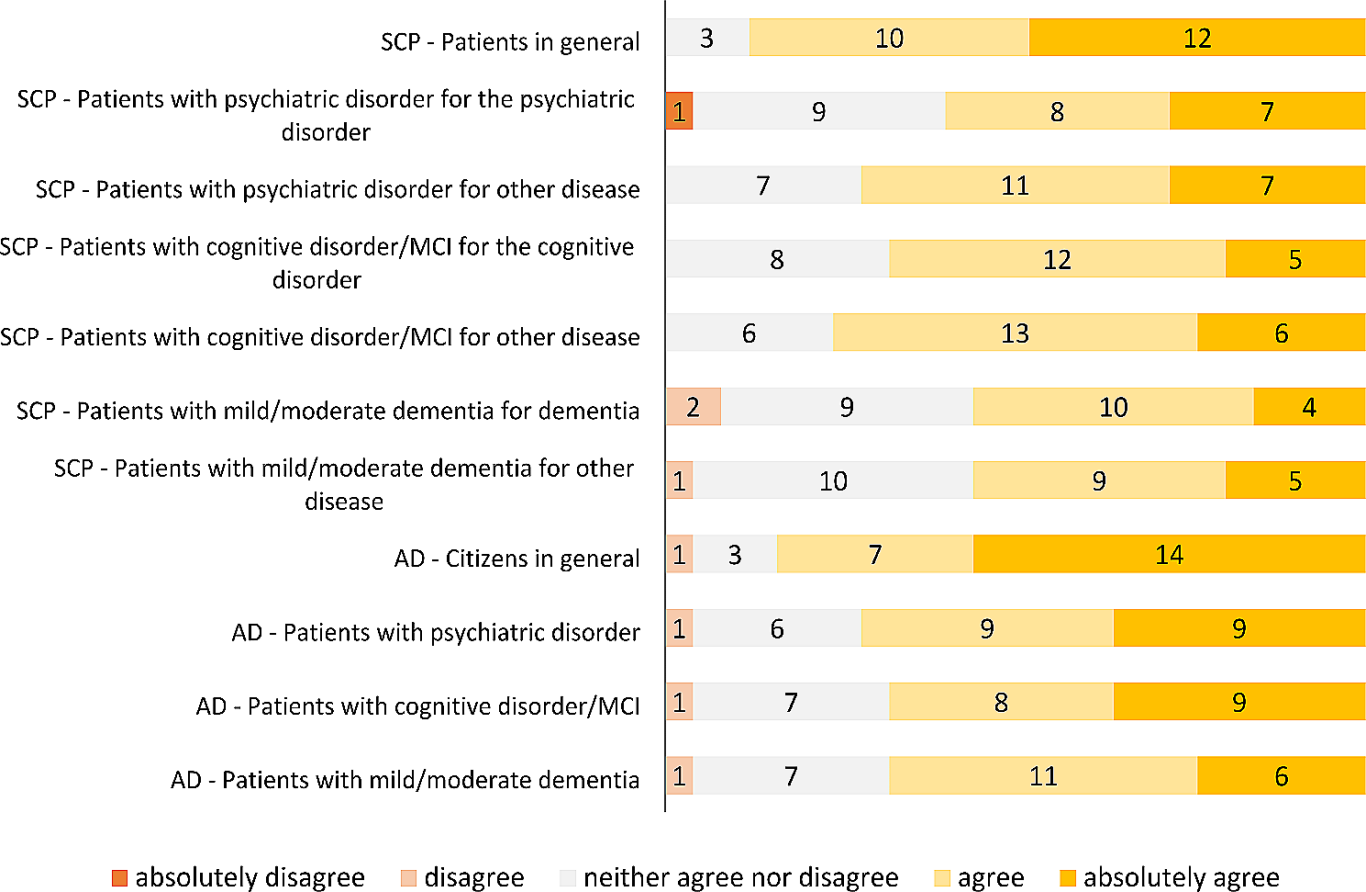
Physicians’ agreement on the utility of realizing SCP and writing AD in the three patient groups

v) Opinion on the utility of SCP and AD for patients with psychiatric disorders, cognitive disorders, mild-moderate dementia. The respondents’ opinions on the utility of realizing SCP for patients in general, for patients with psychiatric disorders, cognitive disorders and dementia (for their specific disorders or other health condition), and on the utility of writing AD for citizens in general, and for patients with psychiatric disorders, cognitive disorders and dementia are reported in Fig. 3.

vi) Opinion on the importance of the trusted person. The large majority of physicians believes it is important to have a trusted person for patients in general (7 absolutely agree, 14 agree, 4 neutral), and for patients with psychiatric disorders, cognitive disorders and dementia (for all these groups: 9 absolutely agree, 12 agree, 4 neutral).

vii) Personal experience as clinician over SCP. A section of the survey was dedicated to SCP. We asked physicians whether in their medical practice they introduced the theme of SCP with one or more patients (without then effectively arranging for SCP), and whether they arranged for SCP. Thirteen physicians neither introduced the theme nor arranged for SCP; 2 have only introduced the topic without ever having arranged for SCP; 4 have both just introduced the theme and arranged for SCP; and 6 arranged for SCP without never only introducing the topic.

The 19 physicians who did not just introduce the theme of SCP did not do so for the following reasons: no patient was in clinical condition to require the discussion (12); not my responsibility to discuss the topic (4); the patient was unable to understand the SCP process (2); the patient did not have sufficient knowledge of the diagnosis/prognosis (1); due to reservations from family members/relatives (1); due to lack of time (1).

The 15 physicians who never realized SCP did not do so for the following reasons: no patient was in clinical condition to require SCP (9); not my responsibility (4); reservations of family members/relatives (2); the patient did not have sufficient knowledge of the diagnosis/prognosis (1); the patient was unable to understand the SCP process (1). No physician did not introduce the theme or did not arrange for SCP due to uncertainty about when to start the discussion/the planning or because of lack of interest on the part of the patient.

Physicians who introduced the theme (6, of whom 3 psychiatrists and 3 geriatricians) did so between 1 and 2 times (3) or more than 20 times (3), by patient initiative (5) by request of the relatives (4) on their own initiative (3). Physicians who arranged for SCP (10, of whom 8 psychiatrists and 2 geriatricians) did so between 1 and 2 times (3), 3 and 5 times (1), 6 and 10 times (2), 11 and 20 times (1) and more than 20 times (3), by their own initiative (6), by patient initiative (3), by request of the relatives (3), or following institutional mandate (1).

Patients who benefit from both introduction of the theme and effective realization of SCP fell into all the groups of our interest (patients suffering from psychiatric disorder with or without another chronic and disabling disease or disease characterized by an inevitable progression with unfavourable prognosis, cognitive disorder/mild cognitive impairment (MCI) with or without other disease, dementia with or without other disease), plus one patient with another pathology.

When SCP was realized, this was done within a dedicated meeting (6) within a scheduled visit (3) or as a specialist consultation (1); on average in one time (4), in 2–3 times (1), in 4–5 times (1), in more than 5 times (4). Two physicians arranged SCP exclusively with the patient; 3 physicians without the patient (just with family member with or without other team members); 5 physicians realized SCP in the presence of multiple stakeholders: the patient and family members and/or trusted person (3), the previous plus other health professionals (2).

SCP was tracked by completing a section of the medical record (5), by writing a specific report (3), orally (1), as part of a consultation (1). In no case were a video recording or other aids used. SCP arrangement was communicated to the institution care team (3), or family physician (2), or a territorial care team and other specialists (2), or a combination of the previous (2); one respondent (who realized oral SCP) communicated just to patient or family members.

Five physicians assisted patients suffering from psychiatric disorder, cognitive disorder and dementia with whom other colleagues had previously arranged SCP, between 1 and 2 times (3) or more than 20 times (2). Four physicians were asked (in two cases more than 20 times) to participate in SCP promoted by a colleague.

viii) Personal experience as clinician over AD. We concluded the survey with few questions on AD. Five physicians were asked for information/consultation regarding AD (the figure of the trusted person, the AD content and medium), between 1 and 2 times (3), 3 and 5 times (1), 11 and 20 times (1).

Six physicians assisted patients who had written AD, between 1 and 2 times (4), 3 and 5 times (1), 6 and 10 times (1) (the quite large number of people with AD is also due to the presence among them of Jehovah’s witnesses). Finally, 3 physicians promoted SCP for patients who had instead previously written AD, between 1 and 2 times (2) or 6 and 10 times (1).

Open-ended questions. The open-ended questions received very few answers, with some suggestions worth considering. Three respondents would like to have better clarification of the role of physicians in relation to the patient’s will, two question the definition of artificial nutrition and hydration as medical treatment, three physicians would like the law to be brought into clinical reality, including a clarification of the meaning that wordings such as “futile treatment” and “chronic illness with an unfavourable prognosis” assume in the psychiatric field. One raises the question of possible limitations in implementing the law in Catholic facilities.

## Discussion

We conducted the survey four years after the law came into effect in four facilities dedicated to the care of people suffering from psychiatric disorders, cognitive disorders and dementia. Although few surveys were conducted on law n. 219/2017 involving citizens [23], health care professionals [24–29] and Ethics committees [13], we are not aware of any research specifically targeting our patient groups.

About 40% of invited physicians participated in the survey. Just two of them declared poor or very poor knowledge of the law provisions but all would like to learn more. This is an initial finding of the study, namely the perceived need for information and training regarding the provisions of the law, which, given the sample under study, has the potential to translate into better implementation of the tools provided by the norm. This result must also be taken into account when reading other findings of the study, particularly those concerning clinical implementation of the law. Other studies conducted in Italy found a lack of adequate knowledge and understanding of the law among health care professionals, with special regard to the notion of AD and how to translate SCP and AD into practice [24–29]. The need for education and training, whether perceived by respondents or recorded as a result of surveys, is indeed what the different studies on the Italian law have in common. This is understandable as the law introduces a cultural change in the relationship between patients and health professionals that requires time and preparation. Studies in other European countries, such as France, Spain and UK, on law targeting IC, ACP, AD also observed mixed knowledge of these instruments and underlined the need for widespread education [29].

Although over one third is neutral, the majority of physicians showed to share the spirit of the law that focuses on subject’s right to self-determination in a context of trust relationship with the medical doctors. Neutrality decreases and agreement increases on the single provisions of the law, including the three tools – IC, SCP, AD - proposed by the legislature to enable individuals to exercise their right of choice in healthcare matters. A positive opinion on the law regarded as a legal instrument able both to safeguard patient’s self-determination and support professionals in providing care was also found in other studies conducted among Italian healthcare professionals [25, 27, 28].

Given the long-running debate on the law, where different ethical perspectives were confronted, we collected data on the participants’ religious belief as it would have been interesting to compare possible differences in agreement with the law between believers and non-believers, but the small number of people claiming to be atheists/agnostics does not permit adequately powered statistical comparisons.

One important result regards the clarity of the concept of capacity in the law in view of its application in clinical practice: the question received mixed answers with just half of the sample agreeing on its clarity. This may impact on the implementation of the law in clinical practice and risks to introduce heterogeneity or even inequality among subjects. The circumstance that psychiatrists and geriatricians doubt the clarity of the concept makes the result even more significant, as these are the physicians who most work with individuals who may experience impaired capacity. The concept of capacity in law n. 219/2017 has actually been widely discussed [21, 30–35]. The law aims at enabling the exercise of the right to therapeutic self-determination and ensuring its best expression even for people without legal or natural capacity. Nevertheless, some criticalities which affect the wording of the law - characterised by a heterogeneous use of the terms referring to capacity and by their interpretability - show the difficulty of reconciling the rigidity of the legal categories of capacity with the complex and changing nature of the clinical conditions and asks for possible correctives from both the healthcare contexts and legal practitioners aiming at achieving maximum approximation between the formal plan of the law and the real contexts of care [21]. Law n. 219/2017 does not specify criteria for determining a person’s capacity, nor the manner, timing and healthcare professionals in charge of the assessment. These aspects, moreover, are not regulated in any other Italian legal tool. They should therefore be addressed within clinical settings, taking into consideration the subtle and complex ways in which mental disorders can affect capacity. A focus on the intellectual and cognitive abilities of the patient, for instance, fails to capture the range of complex difficulties affecting decision making, such as shift in values and changes in the sense of personal identity, observed in people with disorders such as anorexia nervosa [36]. Instead, the analysis of intellectual and cognitive abilities may seem more appropriate in dementias, such as Alzheimer’s disease, but it is still not sufficient to capture the complexity of the person’s clinical and existential situation and his/her capacity to make decisions [31].

Uncertainty about how to interpret the concept of capacity in law n. 219/2017 may partially justify the high number of respondents (from 10 to 15 for each item) who neither agree nor disagree on the legitimacy in principle to realize SCP and AD by patients suffering from psychiatric disorders, cognitive disorders and especially mild to moderate dementia. In any case, the result is rather surprising because the law in no way excludes in principle these categories of individuals from exercising their right to self-determination through the use of the tools provided by the legislation. Once again, the main reason for these responses seems to be found in the difficulty for physicians to drop the formality of the law into the specificity of clinical situations. While in fact these patients are in principle legitimized by the law to realize advance planning, the factual possibility to make health related choices in advance is dependent on their specific situation where a mixture of the forms of incompetence and legal incapacity may occur and the person’s factual situation may not coincide perfectly with her/his legal condition [31]. Participants’ responses showed that the topic deserves clarification and further discussion. This is especially true for the psychiatric field that comprises a number of very different clinical conditions and where the issue of planning in advance has been less explored than it has been for the area of major cognitive disorders where advance planning, including access to palliative care, is better framed [37, 38].

The agreement on the usefulness of SCP and AD for our patient groups is considerably higher than agreement on the legitimacy of their use, with the clear majority of respondents who recognizes the utility of the tools for each group and situation investigated (from 14 to 19 agree/absolutely agree for SCP and from 17 to 18 agree/absolutely agree for AD). Nevertheless, agreement on utility is markedly lower than the one acknowledged for patients and citizens in general. Interestingly, respondents consider SCP and AD more useful to our patient groups for planning other than psychiatric and cognitive conditions and less useful for patients with dementia than for the other two groups. This is little understandable especially for care planning in MCI and dementia, which may be considered ideal situations to plan care in advance. In fact, SCP is primarily a tool for planning a “biphasic” care pathway that begins with a patient able to interact directly with health care professionals and operates even when the patient loses all or part of this ability [39], which is also the case of patients suffering from dementia. Indeed, a number of papers underline the importance to offer people living with dementia the opportunity to discuss advance care plan [37, 40–42], to respect subjects’ wishes, preferences, beliefs and values regarding their future care. One possible explanation for our findings could be that respondents consider people with cognitive disorders still not in a situation to deserve SCP discussion and people with dementia not able anymore to be involved in SCP. The time - not too early but not too late - when to start SCP in dementia is in fact a crucial element, as it may vary from case to case and situation to situation [31, 43]. Anyway, authoritative perspectives consider that physicians should start ACP discussion “as soon as the diagnosis is made, when the patient can still be actively involved” [37] as “it always seems early until it is too late” [44].

There is broad agreement on the importance for patients of appointing a trusted person in making SCP or AD. This gives reason for the respondents’ interest in learning more about the role of the trusted person, which is indeed an important opportunity provided by the law [12, 45].

Coming to clinical practice, we considered of particular interest to investigate into SCP implementation: SCP is in fact an extraordinary tool for care personalization and respect for the patient’s self-determination. Half of the physicians never introduced the theme nor arranged for SCP and the main reason for this was that no patient was in a clinical situation to require it. Interestingly, physicians who introduced the theme did it more because of patients’ and relatives’ request than by their own initiative, while those who arranged for SCP did so equally by their own initiative and by patients’ and relatives’ initiative. This result underlines the role that citizens and patients may have in promoting a medicine that better responds to their own needs and the importance of citizens’ empowerment in relation to health [44, 46]. Indeed, although it is the physician’s responsibility to initiate the discourse on care planning, there is often a complaint of uncertainty about who should initiate it [19, 47], and an informed patient can contribute to the realization of a better care process. The difference in the number of SCP introduced or implemented or participated in per physician (that seems not to be related to the years of experience or patients groups) and the variability in SCP arrangement, in terms of type and number of meetings, mode of tracking and communication, may be due to the specificity of each clinical situation but also to a lack of clear procedure and to some degree of confusion such as that which occurs where respondents declare that SCP is carried out in the absence of the patient or just orally. Even the implementation of SCP for psychiatric patients by three physicians who neither agree nor disagree on its legitimacy underlines the complexity of the issue and the need for further clarification. Interestingly, three physicians had the opportunity to promote SCP for patients who had previously written AD, with the benefit of promoting greater concreteness and adherence to the patient’s specific clinical reality.

### Limitations of the study

Our survey was addressed to four health care facilities belonging to the same organization. This was done for a specific interest in describing the situation of the target facilities and in improving education and better clinical practices if needed. Obviously, this might have had implications in terms of less variability in responses. Moreover, because of the small number of facilities and professionals involved, the study provides preliminary results that we find interesting but in no way claim to be representative of the entire Italian territory. For a broader and more informative picture of the situation regarding the implementation of the law for patients with psychiatric disorders, cognitive disorders and dementia, the promotion of similar surveys in other Italian health care facilities would be advisable.

## Conclusions

Overall, our survey registered a need for more clarity regarding the interpretation and implementation of the law in our patient groups. There are in particular two related areas that deserve further discussion and clarification across ethical, legal and clinical context: the first concerns the question of whether these patient groups are in principle legitimized by the law to realize SCP or write AD (that also includes the question about what falls within the definition of “chronic and disabling disease or disease characterized by an inevitable progression with unfavourable prognosis” provided by the law); the second concerns the notion of capacity required by the law and how this notion can be declined in real-life situations.

Specific training carried out through a discussion between jurists and clinicians on these issues and the proposal of shared good practices could help in realizing a care that is both equitable among subjects and better for the single individual.

### Electronic supplementary material

Below is the link to the electronic supplementary material.

Supplementary Material 1

Supplementary Material 2

Supplementary Material 3

## Data Availability

The questionnaire used for the survey is available as supplementary material in the original Italian version and English translation. The dataset with responses to the questionnaire is available as supplementary material.

## Abbreviations

ACP: Advance Care Planning
AD: Advance Directives
IC: Informed Consent
IRCCS: Italian Institute for Research and Care
MCI: Mild Cognitive Impairment
SCP: Shared Care Planning
