# Supplementary material for: Towards the implementation of law n. 219/2017 on informed consent and advance directives for patients with psychiatric disorders and dementia. Physicians’ knowledge, attitudes and practices in four northern Italian health care facilities
Source: BMC Med Ethics. 2024 Jan 6;25:7. doi: 10.1186/s12910-023-00997-8 (PMC10771689; doi:10.1186/s12910-023-00997-8)
Supplement: Supplementary file 1 — Supplementary Material 1 [file 12910_2023_997_MOESM1_ESM.docx]

**Questionario Medici e Legge 219/2017**

* Campo obbligatorio

1) Come valuta la sua conoscenza della legge n. 219/2017 su Norme in materia di consenso

informato e di disposizioni anticipate di trattamento? *

molto povera

povera

né povera né buona

buona

molto buona

2) Come si è informato e formato riguardo alla legge? [è possibile più di una risposta] *

mass media

ricerca in internet

riviste scientifiche

iniziative di formazione organizzate dalla struttura sanitaria dove lavoro

iniziative di formazione organizzate da altre strutture sanitarie

iniziative di formazione organizzate da organismi non sanitari

altro: _________________________________________________________________________________

3) Ritiene di volersi informare/formare maggiormente sulla legge 219/2017? *

assolutamente no

no

né sì né no

sì

assolutamente sì

4) Quale aspetto della legge vorrebbe approfondire? *

a) La comunicazione con il paziente e il consenso informato *

assolutamente in disaccordo

in disaccordo

né d’accordo né in disaccordo

d’accordo

assolutamente d’accordo

b) Le cure palliative e la dignità nella fase finale della vita *

assolutamente in disaccordo

in disaccordo

né d’accordo né in disaccordo

d’accordo

assolutamente d’accordo

c) Il caso dei pazienti minori di età *

assolutamente in disaccordo

in disaccordo

né d’accordo né in disaccordo

d’accordo

assolutamente d’accordo

d) Il caso dei pazienti in condizioni di incapacità *

assolutamente in disaccordo

in disaccordo

né d’accordo né in disaccordo

d’accordo

assolutamente d’accordo

e) Il ruolo del fiduciario, della persona di fiducia e dei famigliari *

assolutamente in disaccordo

in disaccordo

né d’accordo né in disaccordo

d’accordo

assolutamente d’accordo

f) La pianificazione condivisa delle cure *

assolutamente in disaccordo

in disaccordo

né d’accordo né in disaccordo

d’accordo

assolutamente d’accordo

g) Le disposizioni anticipate di trattamento *

assolutamente in disaccordo

in disaccordo

né d’accordo né in disaccordo

d’accordo

assolutamente d’accordo

5) Quale è complessivamente il suo grado di accordo riguardo al dettato della legge? *

assolutamente in disaccordo

in disaccordo

né d’accordo né in disaccordo

d’accordo

assolutamente d’accordo

6) Quale è il suo grado di accordo relativamente ai singoli dettati della legge?

a) Consenso informato (Articolo 1; ad eccezione degli aspetti relativi agli obblighi organizzativi e di formazione di cui ai commi 8-10) *

assolutamente in disaccordo

in disaccordo

né d’accordo né in disaccordo

d’accordo

assolutamente d’accordo

Ci sono aspetti che vorrebbe cambiare?

Se sì, quali ______________________________________________________________________________

Se sì, in che modo ________________________________________________________________________

b) Obblighi organizzativi e di formazione (Articolo 1, commi 8-10) *

assolutamente in disaccordo

in disaccordo

né d’accordo né in disaccordo

d’accordo

assolutamente d’accordo

Ci sono aspetti che vorrebbe cambiare?

Se sì, quali ______________________________________________________________________________

Se sì, in che modo ________________________________________________________________________

c) Terapia del dolore, divieto di ostinazione irragionevole delle cure e dignità nella fase finale della vita (Articolo 2) *

assolutamente in disaccordo

in disaccordo

né d’accordo né in disaccordo

d’accordo

assolutamente d’accordo

Ci sono aspetti che vorrebbe cambiare?

Se sì, quali ______________________________________________________________________________

Se sì, in che modo ________________________________________________________________________

d) Minori (Articolo 3) *

assolutamente in disaccordo

in disaccordo

né d’accordo né in disaccordo

d’accordo

assolutamente d’accordo

Ci sono aspetti che vorrebbe cambiare?

Se sì, quali ______________________________________________________________________________

Se sì, in che modo ________________________________________________________________________

e) Persone incapaci (Articolo 3) *

assolutamente in disaccordo

in disaccordo

né d’accordo né in disaccordo

d’accordo

assolutamente d’accordo

Ci sono aspetti che vorrebbe cambiare?

Se sì, quali ______________________________________________________________________________

Se sì, in che modo ________________________________________________________________________

f) Disposizioni anticipate di trattamento (DAT) (Articolo 4) *

assolutamente in disaccordo

in disaccordo

né d’accordo né in disaccordo

d’accordo

assolutamente d’accordo

Ci sono aspetti che vorrebbe cambiare?

Se sì, quali ______________________________________________________________________________

Se sì, in che modo ________________________________________________________________________

g) Pianificazione condivisa delle cure (PCC) (Articolo 5) *

assolutamente in disaccordo

in disaccordo

né d’accordo né in disaccordo

d’accordo

assolutamente d’accordo

Ci sono aspetti che vorrebbe cambiare?

Se sì, quali ______________________________________________________________________________

Se sì, in che modo ________________________________________________________________________

7) Da medico specialista nel disturbo psichiatrico/disturbo cognitivo/demenze, ritiene che la legge affronti il tema della capacità/incapacità del soggetto in modo adeguato nella prospettiva di una applicazione sufficientemente chiara nella pratica? *

assolutamente in disaccordo

in disaccordo

né d’accordo né in disaccordo

d’accordo

assolutamente d’accordo

Se assolutamente in disaccordo/in disaccordo, quali aspetti dovrebbero essere chiariti?

8) Ritiene che in linea di principio le persone con disturbo psichiatrico siano giuridicamente legittimate, in base alla legge n. 219/2017, a realizzare una pianificazione condivisa delle cure, per la patologia psichiatrica? *

assolutamente in disaccordo

in disaccordo

né d’accordo né in disaccordo

d’accordo

assolutamente d’accordo

Osservazioni aggiuntive:

9) Ritiene che in linea di principio le persone con disturbo psichiatrico siano giuridicamente legittimate, in base alla legge n. 219/2017, a realizzare una pianificazione condivisa delle cure, per altra malattia cronica e invalidante o caratterizzata da una inarrestabile evoluzione con prognosi infausta? *

assolutamente in disaccordo

in disaccordo

né d’accordo né in disaccordo

d’accordo

assolutamente d’accordo

Osservazioni aggiuntive:

10) Ritiene che in linea di principio le persone con disturbo psichiatrico siano giuridicamente legittimate, in base alla legge n. 219/2017, a redigere le disposizioni anticipate di trattamento? *

assolutamente in disaccordo

in disaccordo

né d’accordo né in disaccordo

d’accordo

assolutamente d’accordo

Osservazioni aggiuntive:

11) Ritiene che in linea di principio le persone con disturbo cognitivo/MCI siano giuridicamente legittimate, in base alla legge n. 219/2017, a realizzare una pianificazione condivisa delle cure, per il disturbo cognitivo? *

assolutamente in disaccordo

in disaccordo

né d’accordo né in disaccordo

d’accordo

assolutamente d’accordo

Osservazioni aggiuntive:

12) Ritiene che in linea di principio le persone con disturbo cognitivo/MCI siano giuridicamente legittimate, in base alla legge n. 219/2017, a realizzare una pianificazione condivisa delle cure, per altra malattia cronica e invalidante o caratterizzata da una inarrestabile evoluzione con prognosi infausta? *

assolutamente in disaccordo

in disaccordo

né d’accordo né in disaccordo

d’accordo

assolutamente d’accordo

Osservazioni aggiuntive:

13) Ritiene che in linea di principio le persone con disturbo cognitivo/MCI siano giuridicamente legittimate, in base alla legge n. 219/2017, a redigere le disposizioni anticipate di trattamento? *

assolutamente in disaccordo

in disaccordo

né d’accordo né in disaccordo

d’accordo

assolutamente d’accordo

Osservazioni aggiuntive:

14) Ritiene che in linea di principio le persone con demenza lieve/moderata siano giuridicamente legittimate, in base alla legge n. 219/2017, a realizzare una pianificazione condivisa delle cure, per la demenza? *

assolutamente in disaccordo

in disaccordo

né d’accordo né in disaccordo

d’accordo

assolutamente d’accordo

Osservazioni aggiuntive:

15) Ritiene che in linea di principio le persone con demenza lieve/moderata siano giuridicamente legittimate, in base alla legge n. 219/2017, a realizzare una pianificazione condivisa delle cure, per altra malattia cronica e invalidante o caratterizzata da una inarrestabile evoluzione con prognosi infausta? *

assolutamente in disaccordo

in disaccordo

né d’accordo né in disaccordo

d’accordo

assolutamente d’accordo

Osservazioni aggiuntive:

16) Ritiene che in linea di principio le persone con demenza lieve/moderata siano giuridicamente legittimate, in base alla legge n. 219/2017, a redigere le disposizioni anticipate di trattamento? *

assolutamente in disaccordo

in disaccordo

né d’accordo né in disaccordo

d’accordo

assolutamente d’accordo

Osservazioni aggiuntive:

17) Per i pazienti in generale, ritiene utile la predisposizione di una pianificazione condivisa delle cure? *

assolutamente in disaccordo

in disaccordo

né d’accordo né in disaccordo

d’accordo

assolutamente d’accordo

Perché:

18) Nel caso di paziente con disturbo psichiatrico, ritiene/riterrebbe utile la predisposizione di una pianificazione condivisa delle cure, per la patologia psichiatrica? *

assolutamente in disaccordo

in disaccordo

né d’accordo né in disaccordo

d’accordo

assolutamente d’accordo

Perché:

19) Nel caso di paziente con disturbo psichiatrico, ritiene/riterrebbe utile la predisposizione di una pianificazione condivisa delle cure, per altra malattia cronica e invalidante o caratterizzata da una inarrestabile evoluzione con prognosi infausta? *

assolutamente in disaccordo

in disaccordo

né d’accordo né in disaccordo

d’accordo

assolutamente d’accordo

Perché:

20) Nel caso di paziente con disturbo cognitivo/MCI ritiene/riterrebbe utile la predisposizione di una pianificazione condivisa delle cure, per il disturbo cognitivo? *

assolutamente in disaccordo

in disaccordo

né d’accordo né in disaccordo

d’accordo

assolutamente d’accordo

Perché:

21) Nel caso di paziente con disturbo cognitivo/MCI ritiene/riterrebbe utile la predisposizione di una pianificazione condivisa delle cure, per altra malattia cronica e invalidante o caratterizzata da una inarrestabile evoluzione con prognosi infausta? *

assolutamente in disaccordo

in disaccordo

né d’accordo né in disaccordo

d’accordo

assolutamente d’accordo

Perché:

22) Nel caso di paziente con demenza lieve/moderata ritiene/riterrebbe utile la predisposizione di una pianificazione condivisa delle cure, per la demenza? *

assolutamente in disaccordo

in disaccordo

né d’accordo né in disaccordo

d’accordo

assolutamente d’accordo

Perché:

23) Nel caso di paziente con demenza lieve/moderata ritiene/riterrebbe utile la predisposizione di una pianificazione condivisa delle cure, per altra malattia cronica e invalidante o caratterizzata da una inarrestabile evoluzione con prognosi infausta? *

assolutamente in disaccordo

in disaccordo

né d’accordo né in disaccordo

d’accordo

assolutamente d’accordo

Perché:

24) Per i cittadini in generale, ritiene utile la predisposizione delle DAT? *

assolutamente in disaccordo

in disaccordo

né d’accordo né in disaccordo

d’accordo

assolutamente d’accordo

Perché:

25) Nel caso di persone con disturbo psichiatrico, ritiene/riterrebbe utile la predisposizione delle DAT? *

assolutamente in disaccordo

in disaccordo

né d’accordo né in disaccordo

d’accordo

assolutamente d’accordo

Perché:

26) Nel caso di persone con disturbo cognitivo/MCI ritiene/riterrebbe utile la predisposizione delle DAT? *

assolutamente in disaccordo

in disaccordo

né d’accordo né in disaccordo

d’accordo

assolutamente d’accordo

Perché: _______________________________________________________________________________________

27) Nel caso di persone con demenza lieve/moderata ritiene/riterrebbe utile la predisposizione delle DAT? *

assolutamente in disaccordo

in disaccordo

né d’accordo né in disaccordo

d’accordo

assolutamente d’accordo

Perché:

28) Per i cittadini e pazienti in generale, ritiene importante la presenza di un fiduciario? *

assolutamente in disaccordo

in disaccordo

né d’accordo né in disaccordo

d’accordo

assolutamente d’accordo

Perché:

29) Per le persone con disturbo psichiatrico, ritiene importante la presenza di un fiduciario? *

assolutamente in disaccordo

in disaccordo

né d’accordo né in disaccordo

d’accordo

assolutamente d’accordo

Perché:

30) Per le persone con disturbo cognitivo/MCI, ritiene importante la presenza di un fiduciario? *

assolutamente in disaccordo

in disaccordo

né d’accordo né in disaccordo

d’accordo

assolutamente d’accordo

Perché:

31) Per le persone con demenza, ritiene importante la presenza di un fiduciario? *

assolutamente in disaccordo

in disaccordo

né d’accordo né in disaccordo

d’accordo

assolutamente d’accordo

Perché:

32) Nella sua attività di medico, si è trovato nella situazione di introdurre il tema della pianificazione condivisa delle cure con uno o più pazienti, senza che, poi, sia stata effettivamente predisposta una PCC? *

Sì

No

33) Se non ha introdotto il tema della PCC, perché: [è possibile più di una risposta] *

Non è di mia competenza discutere il tema della PCC

Nessun paziente era in una condizione clinica da richiedere la discussione

Il paziente non aveva sufficiente conoscenza della diagnosi/prognosi

Il paziente non era in grado di comprendere il processo di PCC

Ho proposto la discussione al paziente, che non era interessato

Per mancanza di tempo

Per incertezza su quando iniziare la discussione

Per riserve da parte dei famigliari/congiunti

Altro: _________________________________________________________________________________

34) Se sì, quante volte? *

Tra 1 e 2

Tra 3 e 5

Tra 6 e 10

Tra 11 e 20

Oltre 20

35) Se sì, su iniziativa di: [è possibile più di una risposta] *

Su mia iniziativa

Su iniziativa del paziente

Su richiesta dei congiunti

Altro: _________________________________________________________________________________

36) Se sì, quale era la diagnosi/condizione clinica dei pazienti? [è possibile più di una risposta] ^*^

Paziente con disturbo psichiatrico

Paziente con disturbo psichiatrico in presenza di altra malattia cronica e invalidante o caratterizzata da una inarrestabile evoluzione con prognosi infausta

Paziente con disturbo cognitivo/MCI

Paziente con disturbo cognitivo/MCI in presenza di altra malattia cronica e invalidante o caratterizzata da una inarrestabile evoluzione con prognosi infausta

Paziente con demenza

Paziente con demenza in presenza di altra malattia cronica e invalidante o caratterizzata da una inarrestabile evoluzione con prognosi infausta

Altro: _________________________________________________________________________________

37) Nella sua attività di medico, si è trovato nella situazione di predisporre una pianificazione

condivisa delle cure con uno o più pazienti? *

Sì

No

38) Se no, perché: [è possibile più di una risposta] *

Non è di mia competenza predisporre una PCC

Nessun paziente era in una condizione clinica da richiedere la PCC

Il paziente non aveva sufficiente conoscenza della diagnosi/prognosi

Il paziente non era in grado di comprendere il processo di PCC

Ho proposto la PCC al paziente, che ha rifiutato

Per mancanza di tempo

Per incertezza su quando iniziare il percorso

Per riserve da parte dei famigliari/congiunti

Altro: _________________________________________________________________________________

39) Se sì, quante volte? *

Tra 1 e 2

Tra 3 e 5

Tra 6 e 10

Tra 11 e 20

Oltre 20

40) Se sì, su iniziativa di: [è possibile più di una risposta] *

Su mia iniziativa

Su iniziativa del paziente

Su richiesta dei congiunti

Altro: _________________________________________________________________________________

41) Se sì, quale era la diagnosi/condizione clinica dei pazienti? [è possibile più di una risposta] *

Paziente con disturbo psichiatrico

Paziente con disturbo psichiatrico in presenza di altra malattia cronica e invalidante o caratterizzata da una inarrestabile evoluzione con prognosi infausta

Paziente con disturbo cognitivo/MCI

Paziente con disturbo cognitivo/MCI in presenza di altra malattia cronica e invalidante o caratterizzata da una inarrestabile evoluzione con prognosi infausta

Paziente con demenza

Paziente con demenza in presenza di altra malattia cronica e invalidante o caratterizzata da una inarrestabile evoluzione con prognosi infausta

Altro: _________________________________________________________________________________

42) Se sì, in quale spazio operativo prevalente ha informato il paziente e predisposto la PCC? *

Visita programmata ad altro fine

Colloquio dedicato

Altro: _________________________________________________________________________________

43) Se sì, chi era presente durante l’informazione e/o la predisposizione della PCC? [è possibile più di una risposta] *

Paziente

Fiduciario

Famigliare/congiunto

Personale dell’equipe

Altro: _________________________________________________________________________________

44) Se sì, in media, quanti sono stati i momenti dedicati alla informazione e predisposizione della PCC? *

1

2-3

4-5

oltre i 5

Altro: _________________________________________________________________________________

45) Se sì, come ha tracciato la PCC? *

Ho elaborato una relazione sul caso specifico

Ho compilato una sezione della cartella clinica

Ho utilizzato un form ad hoc per la PCC

Altro: _________________________________________________________________________________

46) Se sì, ha utilizzato la videoregistrazione o altri ausili per la comunicazione? *

Sì

No

47) Se sì, a chi è stata comunicata la predisposizione della PCC? [è possibile più di una risposta] *

Medico di famiglia

Altro specialista

Équipe di cura dell’istituzione

Team di cura territoriale

Altro: _________________________________________________________________________________

48) Nella sua attività di medico, ha incontrato pazienti con i quali altri colleghi avevano in precedenza predisposto una pianificazione condivisa delle cure? *

Sì

No

49) Se sì, quante volte? *

Tra 1 e 2

Tra 3 e 5

Tra 6 e 10

Tra 11 e 20

Oltre 20

50) Se sì, quale era la condizione clinica dei pazienti? [è possibile più di una risposta] *

Paziente con disturbo psichiatrico

Paziente con disturbo psichiatrico in presenza di altra malattia cronica e invalidante o caratterizzata da una inarrestabile evoluzione con prognosi infausta

Paziente con disturbo cognitivo/MCI

Paziente con disturbo cognitivo/MCI in presenza di altra malattia cronica e invalidante o caratterizzata da una inarrestabile evoluzione con prognosi infausta

Paziente con demenza

Paziente con demenza in presenza di altra malattia cronica e invalidante o caratterizzata da una inarrestabile evoluzione con prognosi infausta

Altro: _________________________________________________________________________________

51) Nella sua attività di medico, le è stato chiesto di partecipare alla predisposizione o realizzazione di una pianificazione condivisa delle cure promossa da un collega? *

Sì

No

52) Se sì, quante volte? *

Tra 1 e 2

Tra 3 e 5

Tra 6 e 10

Tra 11 e 20

Oltre 20

53) Se sì, su iniziativa di: [è possibile più di una risposta] *

Su iniziativa di un collega medico

Su iniziativa del paziente

Su richiesta dei congiunti

Altro: _________________________________________________________________________________

54) Se sì, quale era la condizione clinica dei pazienti? [è possibile più di una risposta] *

Paziente con disturbo psichiatrico

Paziente con disturbo psichiatrico in presenza di altra malattia cronica e invalidante o caratterizzata da una inarrestabile evoluzione con prognosi infausta

Paziente con disturbo cognitivo/MCI

Paziente con disturbo cognitivo/MCI in presenza di altra malattia cronica e invalidante o caratterizzata da una inarrestabile evoluzione con prognosi infausta

Paziente con demenza

Paziente con demenza in presenza di altra malattia cronica e invalidante o caratterizzata da una inarrestabile evoluzione con prognosi infausta

Altro: _________________________________________________________________________________

55) Nella sua attività di medico, ha incontrato pazienti che le hanno chiesto informazioni/consulenza riguardo alla redazione delle disposizioni anticipate di trattamento? *

Sì

No

56) Se sì, quante volte? *

Tra 1 e 2

Tra 3 e 5

Tra 6 e 10

Tra 11 e 20

Oltre 20

57) Se sì, riguardo a quale aspetto: [è possibile più di una risposta] *

Aspetti relativi al contenuto

Ruolo del fiduciario

Modalità di redazione e deposito

Rispetto delle disposizioni da parte del medico

Altro: _________________________________________________________________________________

58) Se sì, quale era la condizione clinica dei pazienti? [è possibile più di una risposta] *

Paziente con disturbo psichiatrico

Paziente con disturbo psichiatrico in presenza di altra malattia cronica e invalidante o caratterizzata da una inarrestabile evoluzione con prognosi infausta

Paziente con disturbo cognitivo/MCI

Paziente con disturbo cognitivo/MCI in presenza di altra malattia cronica e invalidante o caratterizzata da una inarrestabile evoluzione con prognosi infausta

Paziente con demenza

Paziente con demenza in presenza di altra malattia cronica e invalidante o caratterizzata da una inarrestabile evoluzione con prognosi infausta

Altro: _________________________________________________________________________________

59) Nella sua attività di medico, ha incontrato pazienti che avevano redatto le disposizioni anticipate di trattamento? *

Sì

No

60) Se sì, quante volte? *

Tra 1 e 2

Tra 3 e 5

Tra 6 e 10

Tra 11 e 20

Oltre 20

61) Se sì, quale era la condizione clinica dei pazienti? [è possibile più di una risposta] *

Paziente con disturbo psichiatrico

Paziente con disturbo psichiatrico in presenza di altra malattia cronica e invalidante o caratterizzata da una inarrestabile evoluzione con prognosi infausta

Paziente con disturbo cognitivo/MCI

Paziente con disturbo cognitivo/MCI in presenza di altra malattia cronica e invalidante o caratterizzata da una inarrestabile evoluzione con prognosi infausta

Paziente con demenza

Paziente con demenza in presenza di altra malattia cronica e invalidante o caratterizzata da una inarrestabile evoluzione con prognosi infausta

Altro: _________________________________________________________________________________

62) Nella sua attività di medico, ha incontrato pazienti che avevano in precedenza redatto disposizioni anticipate di trattamento con i quali ha invece promosso una pianificazione condivisa delle cure? *

Sì

No

63) Se sì, quante volte? *

Tra 1 e 2

Tra 3 e 5

Tra 6 e 10

Tra 11 e 20

Oltre 20

64) Se sì, quale era la condizione clinica dei pazienti? [è possibile più di una risposta] *

Paziente con disturbo psichiatrico

Paziente con disturbo psichiatrico in presenza di altra malattia cronica e invalidante o caratterizzata da una inarrestabile evoluzione con prognosi infausta

Paziente con disturbo cognitivo/MCI

Paziente con disturbo cognitivo/MCI in presenza di altra malattia cronica e invalidante o caratterizzata da una inarrestabile evoluzione con prognosi infausta

Paziente con demenza

Paziente con demenza in presenza di altra malattia cronica e invalidante o caratterizzata da una inarrestabile evoluzione con prognosi infausta

Altro: _________________________________________________________________________________

65) Osservazioni aggiuntive:

_______________________________________________________________________________________

**Questionnaire Physicians and Law 219/2017**

* Mandatory answer

1) How do you evaluate your knowledge of Law n. 219/2017 “Provisions on informed consent and advance directives”? *

very poor

poor

neither poor nor good

good

very good

2) How did you become informed and educated about the law? [more than one answer is possible] *

Mass media

Internet search

Scientific journals

Training initiatives organised by the health care facility where I work

Training initiatives organised by other health care facilities

Training initiatives organised by non-health care organisations

Other: ________________________________________________________________________________

3) Do you feel you would like to become more informed/educated about Law n. 219/2017? *

Absolutely not

No

Neither yes nor no

Yes

Absolutely yes

4) If absolutely yes/yes, what aspect of the Law would you like to deepen? *

a) Communication with the patient and informed consent

Absolutely disagree

Disagree

Neither agree nor disagree

Agree

Absolutely agree

b) Palliative care and dignity in the end of life *

Absolutely disagree

Disagree

Neither agree nor disagree

Agree

Absolutely agree

c) The case of minor patients *

Absolutely disagree

Disagree

Neither agree nor disagree

Agree

Absolutely agree

d) The case of incapacitated patients *

Absolutely disagree

Disagree

Neither agree nor disagree

Agree

Absolutely agree

e) The role of the trusted person and relatives *

Absolutely disagree

Disagree

Neither agree nor disagree

Agree

Absolutely agree

f) Shared care planning *

Absolutely disagree

Disagree

Neither agree nor disagree

Agree

Absolutely agree

g) Advance directives *

Absolutely disagree

Disagree

Neither agree nor disagree

Agree

Absolutely agree

5) What is your overall degree of agreement regarding the content of the Law? *

Absolutely disagree

Disagree

Neither agree nor disagree

Agree

Absolutely agree

6) What is your degree of agreement regarding the individual provisions of the Law? *

a) Informed consent (Article 1; except for aspects related to organizational and training obligations in paragraphs 8 to 10) *

Absolutely disagree

Disagree

Neither agree nor disagree

Agree

Absolutely agree

Are there any aspects that you would like to change? ____________________________________________

If yes, which ones? ________________________________________________________________________

If yes, how? _____________________________________________________________________________

b) Organizational and training obligations (Article 1, paragraphs 8 to 10) *

Absolutely disagree

Disagree

Neither agree nor disagree

Agree

Absolutely agree

Are there any aspects that you would like to change? ____________________________________________

If yes, which ones? ________________________________________________________________________

If yes, how? _____________________________________________________________________________

c) Pain therapy, prohibition of unreasonable obstinacy in treatment and dignity in the end of life (Article 2) *

Absolutely disagree

Disagree

Neither agree nor disagree

Agree

Absolutely agree

Are there any aspects that you would like to change? ____________________________________________

If yes, which ones? ________________________________________________________________________

If yes, how? _____________________________________________________________________________

d) Minors (Article 3) *

Absolutely disagree

Disagree

Neither agree nor disagree

Agree

Absolutely agree

Are there any aspects that you would like to change? ____________________________________________

If yes, which ones? ________________________________________________________________________

If yes, how? _____________________________________________________________________________

e) Incapacitated adults (Article 3) *

Absolutely disagree

Disagree

Neither agree nor disagree

Agree

Absolutely agree

Are there any aspects that you would like to change? ____________________________________________

If yes, which ones? ________________________________________________________________________

If yes, how? _____________________________________________________________________________

f) Advance directives (Article 4) *

Absolutely disagree

Disagree

Neither agree nor disagree

Agree

Absolutely agree

Are there any aspects that you would like to change? ____________________________________________

If yes, which ones? ________________________________________________________________________

If yes, how? _____________________________________________________________________________

g) Shared care planning (Article 5) *

Absolutely disagree

Disagree

Neither agree nor disagree

Agree

Absolutely agree

Are there any aspects that you would like to change? ____________________________________________

If yes, which ones? ________________________________________________________________________

If yes, how? _____________________________________________________________________________

7) As a physician specialized in psychiatric/cognitive disorder/dementia, do you believe that the Law adequately addresses the topic of the subject's capacity/incapacity in view of a clear application in clinical practice? *

Absolutely disagree

Disagree

Neither agree nor disagree

Agree

Absolutely agree

If absolutely disagree/disagree, which aspects should be clarified?

8) Do you believe that in principle people with psychiatric disorders are legally legitimized under Law n. 219/2017 to realise a shared care planning, for the psychiatric disorder? *

Absolutely disagree

Disagree

Neither agree nor disagree

Agree

Absolutely agree

Additional comments:

9) Do you believe that in principle people with psychiatric disorders are legally legitimized under Law n. 219/2017 to realise a shared care planning, for other chronic and disabling disease or disease characterized by an inevitable progression with unfavourable prognosis? *

Absolutely disagree

Disagree

Neither agree nor disagree

Agree

Absolutely agree

Additional comments:

10) Do you believe that in principle people with psychiatric disorders are legally legitimized under Law n. 219/2017 to write advance directives? *

Absolutely disagree

Disagree

Neither agree nor disagree

Agree

Absolutely agree

Additional comments:

11) Do you believe that in principle people with cognitive disorder/MCI are legally legitimized under Law n. 219/2017 to realise a shared care planning, for the cognitive disorder/MCI? *

Absolutely disagree

Disagree

Neither agree nor disagree

Agree

Absolutely agree

Additional comments:

12) Do you believe that in principle people with cognitive disorder/MCI are legally legitimized under Law n. 219/2017 to realise a shared care planning for other chronic and disabling disease or disease characterized by an inevitable progression with unfavourable prognosis? *

Absolutely disagree

Disagree

Neither agree nor disagree

Agree

Absolutely agree

Additional comments:

13) Do you believe that in principle people with cognitive disorder/MCI are legally legitimized under Law n. 219/2017 to write advance directives? *

Absolutely disagree

Disagree

Neither agree nor disagree

Agree

Absolutely agree

Additional comments:

14) Do you believe that in principle people with mild/moderate dementia are legally legitimized under Law n. 219/2017 to realise a shared care planning for dementia? *

Absolutely disagree

Disagree

Neither agree nor disagree

Agree

Absolutely agree

Additional comments:

15) Do you believe that in principle people with mild/moderate dementia are legally legitimized under Law n. 219/2017 to realise a shared care planning, for other chronic and disabling disease or disease characterized by an inevitable progression with unfavourable prognosis? *

Absolutely disagree

Disagree

Neither agree nor disagree

Agree

Absolutely agree

Additional comments:

16) Do you believe that in principle people with mild/moderate dementia are legally legitimized under Law n. 219/2017 to write advance directives? *

Absolutely disagree

Disagree

Neither agree nor disagree

Agree

Absolutely agree

Additional comments:

17) For patients in general, do you consider the arrangement of a shared care planning useful? *

Absolutely disagree

Disagree

Neither agree nor disagree

Agree

Absolutely agree

Why?

18) In the case of a patient with psychiatric disorder, do you consider/would you consider the arrangement of a shared care planning useful, for the psychiatric disorder? *

Absolutely disagree

Disagree

Neither agree nor disagree

Agree

Absolutely agree

Why?

19) In the case of a patient with psychiatric disorder, do you consider/would you consider the arrangement of a shared care planning useful, for other chronic and disabling disease or disease characterized by an inevitable progression with unfavourable prognosis? *

Absolutely disagree

Disagree

Neither agree nor disagree

Agree

Absolutely agree

Why?

20) In the case of a patient with cognitive disorder/MCI, do you consider/would you consider the arrangement of a shared care planning useful, for the cognitive disorder? *

Absolutely disagree

Disagree

Neither agree nor disagree

Agree

Absolutely agree

Why?

21) In the case of a patient with cognitive disorder/MCI, do you consider/would you consider the arrangement of a shared care planning useful, for other chronic and disabling disease or disease characterized by an inevitable progression with unfavourable prognosis? *

Absolutely disagree

Disagree

Neither agree nor disagree

Agree

Absolutely agree

Why?

22) In the case of a patient with mild/moderate dementia, do you consider/would you consider the arrangement of a shared care planning useful, for dementia? *

Absolutely disagree

Disagree

Neither agree nor disagree

Agree

Absolutely agree

Why?

23) In the case of a patient with mild/moderate dementia, do you consider/would you consider the arrangement of a shared care planning useful, for other chronic and disabling disease or disease characterized by an inevitable progression with unfavourable prognosis? *

Absolutely disagree

Disagree

Neither agree nor disagree

Agree

Absolutely agree

Why?

24) For citizens in general, do you consider the writing of advance directives useful? *

Absolutely disagree

Disagree

Neither agree nor disagree

Agree

Absolutely agree

Why?

25) In the case of people with psychiatric disorder, do you consider/would you consider the writing of advance directives useful? *

Absolutely disagree

Disagree

Neither agree nor disagree

Agree

Absolutely agree

Why?

26) In the case of people with cognitive disorder/MCI, do you consider/would you consider the writing of advance directives useful? *

Absolutely disagree

Disagree

Neither agree nor disagree

Agree

Absolutely agree

Why?

27) In the case of people with mild/moderate dementia, do you consider/would you consider the writing of advance directives useful? *

Absolutely disagree

Disagree

Neither agree nor disagree

Agree

Absolutely agree

Why?

28) For citizens and patients in general, do you consider the presence of a trusted person important? *

Absolutely disagree

Disagree

Neither agree nor disagree

Agree

Absolutely agree

Why?

29) For people with psychiatric disorder, do you consider the presence of a trusted person important? *

Absolutely disagree

Disagree

Neither agree nor disagree

Agree

Absolutely agree

Why?

30) For people with cognitive disorder/MCI, do you consider the presence of a trusted person important? *

Absolutely disagree

Disagree

Neither agree nor disagree

Agree

Absolutely agree

Why?

31) For people with dementia, do you consider the presence of a trusted person important? *

Absolutely disagree

Disagree

Neither agree nor disagree

Agree

Absolutely agree

Why?

32) In your experience as a physician, did you find yourself in the situation of introducing the topic of shared care planning with one or more patients, without then effectively arranging a shared care planning? *

Yes

No

33) If you did not introduce the topic of shared care planning, why? [more than one answer is possible] *

It is not my responsibility to discuss the topic of shared care planning

No patient was in a clinical condition to require the discussion

The patient did not have sufficient knowledge of the diagnosis/prognosis

The patient was unable to understand the process of shared care planning

I proposed the discussion to the patient, who was not interested

Due to lack of time

Due to uncertainty about when to start the discussion

Due to reservations from family members/relatives

Other: ________________________________________________________________________________

34) If yes, how many times? *

Between 1 and 2

Between 3 and 5

Between 6 and 10

Between 11 and 20

More than 20

35) If yes, on whose initiative? [more than one answer is possible] *

On my own initiative

On the patient’s own initiative

At the request of the relatives

Other: ________________________________________________________________________________

36) If yes, what was the diagnosis/clinical condition of the patients? [more than one answer is possible] *

Patient with psychiatric disorder

Patient with psychiatric disorder and other chronic and disabling disease or disease characterized by an inevitable progression with unfavourable prognosis

Patient with cognitive disorder/MCI

Patient with cognitive disorder/MCI and other chronic and disabling disease or disease characterized by an inevitable progression with unfavourable prognosis

Patient with dementia

Patient with dementia and other chronic and disabling disease or disease characterized by an inevitable progression with unfavourable prognosis

Other: ________________________________________________________________________________

37) In your experience as a physician, did you find yourself in the situation of arranging a shared care planning with one or more patients? *

Yes

No

38) If not, why? [more than one answer is possible] *

It is not my responsibility to arrange a shared care planning

No patient was in a clinical condition to require the shared care planning

The patient did not have sufficient knowledge of the diagnosis/prognosis

The patient was unable to understand the process of shared care planning

I proposed the shared care planning to the patient, who refused

Due to lack of time

Due to uncertainty about when to start the discussion

Due to reservations from family members/relatives

Other: ________________________________________________________________________________

39) If yes, how many times? *

Between 1 and 2

Between 3 and 5

Between 6 and 10

Between 11 and 20

More than 20

40) If yes, on whose initiative? [more than one answer is possible] *

On my own initiative

On the patient’s own initiative

At the request of the relatives

Other: ________________________________________________________________________________

41) If yes, what was the diagnosis/clinical condition of the patients? [more than one answer is possible] *

Patient with psychiatric disorder

Patient with psychiatric disorder and other chronic and disabling disease or disease characterized by an inevitable progression with unfavourable prognosis

Patient with cognitive disorder/MCI

Patient with cognitive disorder/MCI and other chronic and disabling disease or disease characterized by an inevitable progression with unfavourable prognosis

Patient with dementia

Patient with dementia and other chronic and disabling disease or disease characterized by an inevitable progression with unfavourable prognosis

Other: ________________________________________________________________________________

42) If yes, mainly in which operational space did you inform the patient and arrange the shared care planning? *

Visit scheduled for another purpose

Specific consultation

Other: ________________________________________________________________________________

43) If yes, who was present during information and/or the arrangement of the shared care planning? [more than one answer is possible] *

Patient

Trusted person

Family member/relative

Members of the healthcare team

Other: ________________________________________________________________________________

44) If yes, how many times on average were dedicated to informing and arranging the shared care planning? *

1

2-3

4-5

More than 5

Other: ________________________________________________________________________________

45) If yes, how did you track the share care planning? *

I prepared a report on the specific case

I completed a section of the medical record

I used an ad hoc form for shared care planning

Other: ________________________________________________________________________________

46) If yes, did you use video recording or other communication aids? *

Yes

No

47) If yes, who was notified of the shared care planning? [more than one answer is possible] *

General practitioner

Other medical specialist

Care team of the facility

Community care team

Other: ________________________________________________________________________________

48) In your experience as a physician, did you meet patients with whom other colleagues had previously arranged a shared care planning? *

Yes

No

49) If yes, how many times? *

Between 1 and 2

Between 3 and 5

Between 6 and 10

Between 11 and 20

More than 20

50) If yes, what was the clinical condition of the patients? [more than one answer is possible] *

Patient with psychiatric disorder

Patient with psychiatric disorder and other chronic and disabling disease or disease characterized by an inevitable progression with unfavourable prognosis

Patient with cognitive disorder/MCI

Patient with cognitive disorder/MCI and other chronic and disabling disease or disease characterized by an inevitable progression with unfavourable prognosis

Patient with dementia

Patient with dementia and other chronic and disabling disease or disease characterized by an inevitable progression with unfavourable prognosis

Other: ________________________________________________________________________________

51) In your experience as a physician, were you asked to participate in the arranging or implementation of a shared care planning promoted by a colleague? *

Yes

No

52) If yes, how many times? *

Between 1 and 2

Between 3 and 5

Between 6 and 10

Between 11 and 20

More than 20

53) If yes, on whose initiative? [more than one answer is possible] *

On the initiative of a medical colleague

On the patient’s initiative

At the request of the relatives

Other: ________________________________________________________________________________

54) If yes, what was the clinical condition of the patients? [more than one answer is possible] *

Patient with psychiatric disorder

Patient with psychiatric disorder and other chronic and disabling disease or disease characterized by an inevitable progression with unfavourable prognosis

Patient with cognitive disorder/MCI

Patient with cognitive disorder/MCI and other chronic and disabling disease or disease characterized by an inevitable progression with unfavourable prognosis

Patient with dementia

Patient with dementia and other chronic and disabling disease or disease characterized by an inevitable progression with unfavourable prognosis

Other: ________________________________________________________________________________

55) In your experience as a physician, did any patients ask you for information/counselling regarding the writing of advance directives? *

Yes

No

56) If yes, how many times? *

Between 1 and 2

Between 3 and 5

Between 6 and 10

Between 11 and 20

More than 20

57) If yes, regarding what aspect? [more than one answer is possible] *

Aspects related to content

Role of trusted person

Mode of writing and deposit

Physician’s compliance with the advance directives

Other: ________________________________________________________________________________

58) If yes, what was the clinical condition of the patients? [more than one answer is possible] *

Patient with psychiatric disorder

Patient with psychiatric disorder and other chronic and disabling disease or disease characterized by an inevitable progression with unfavourable prognosis

Patient with cognitive disorder/MCI

Patient with cognitive disorder/MCI and other chronic and disabling disease or disease characterized by an inevitable progression with unfavourable prognosis

Patient with dementia

Patient with dementia and other chronic and disabling disease or disease characterized by an inevitable progression with unfavourable prognosis

Other: ________________________________________________________________________________

59) In your experience as a physician, did you meet patients who had written advance directives? *

Yes

No

60) If yes, how many times? *

Between 1 and 2

Between 3 and 5

Between 6 and 10

Between 11 and 20

More than 20

61) If yes, what was the clinical condition of the patients? [more than one answer is possible] *

Patient with psychiatric disorder

Patient with psychiatric disorder and other chronic and disabling disease or disease characterized by an inevitable progression with unfavourable prognosis

Patient with cognitive disorder/MCI

Patient with cognitive disorder/MCI and other chronic and disabling disease or disease characterized by an inevitable progression with unfavourable prognosis

Patient with dementia

Patient with dementia and other chronic and disabling disease or disease characterized by an inevitable progression with unfavourable prognosis

Other: ________________________________________________________________________________

62) In your experience as a physician, did you meet patients who had previously written advance directives with whom you have instead promoted a shared care planning? *

Yes

No

63) If yes, how many times? *

Between 1 and 2

Between 3 and 5

Between 6 and 10

Between 11 and 20

More than 20

64) If yes, what was the clinical condition of the patients? [more than one answer is possible] *

Patient with psychiatric disorder

Patient with psychiatric disorder and other chronic and disabling disease or disease characterized by an inevitable progression with unfavourable prognosis

Patient with cognitive disorder/MCI

Patient with cognitive disorder/MCI and other chronic and disabling disease or disease characterized by an inevitable progression with unfavourable prognosis

Patient with dementia

Patient with dementia and other chronic and disabling disease or disease characterized by an inevitable progression with unfavourable prognosis

Other: ________________________________________________________________________________

65) Additional comments:
